# SPR Detection and Discrimination of the Oligonucleotides Related to the Normal and the Hybrid *bcr-abl* Genes by Two Stringency Control Strategies

**DOI:** 10.1186/s11671-016-1226-y

**Published:** 2016-01-13

**Authors:** M. J. Matsishin, Iu. V. Ushenin, A. E. Rachkov, A. P. Solatkin

**Affiliations:** Institute of High Technologies, Taras Shevchenko Kyiv National University, 64 Volodymyrska Str., 01003 Kyiv, Ukraine; Laboratory of Biomolecular Electronics, Institute of Molecular Biology and Genetics of the National Academy of Sciences of Ukraine, 150 Zabolotnogo Str., 03680 Kyiv, Ukraine; V. Ye. Lashkaryov Institute of Semiconductor Physics, NAS of Ukraine, 41, Prospect Nauki, Kyiv, 03028 Ukraine

**Keywords:** SPR, Hybridization biosensor, Stringency control, Ionic strength, Thermodiscrimination

## Abstract

In this study, we applied two stringency control strategies for surface plasmon resonance (SPR) detection of DNA hybridization and discrimination of completely and partially complementary 24-mer sequences. These sequences are specific to the human normal *bcr* and the hybrid *bcr-abl* genes, protein products of which are responsible for some leukemia. SPR sensors based on resonance phenomena in nanoscale gold films are well suited for label-free, real-time investigations of the macromolecule interactions. Thermodynamic parameters obtained using the web server DINAMelt allowed supposing the possibility for realization (a) stringency control based on the ionic strength of the hybridization buffer and (b) stringency control based on the temperature elevation. The first one resulted in that the discrimination index of completely complementary and partially complementary oligonucleotides depending on the target concentration varied from 1.3 to 1.8 in 2 × SSC and from 2.0 to 2.9 in 0.5 × SSC. For implementation of the second stringency control strategy, SPR spectrometer measuring flow cell with built-in high-precision temperature control and regulation as well as corresponding software was created. It is shown that the duplexes formed by the immobilized probes mod-Ph and completely complementary oligonucleotides P1 remained without significant changes until ~50 °C, while the duplexes formed with partially complementary oligonucleotide Bcrex14 almost entirely disrupted at 40 °C. Thus, the absolutely effective thermodiscrimination of this pair of oligonucleotides was achieved in this temperature range (40–50 °C).

## Background

Sequence-specific hybridization between single-stranded oligonucleotides immobilized on a sensor surface and fragments of nucleic acids of the investigated samples is a straightforward way for identification of various genetic and infectious diseases, especially on their early stages. For example, some leukemia can be associated with the so-called Philadelphia chromosome (Ph′ chromosome), which is a result of the reciprocal translocation between human chromosomes 9 and 22 [1, 2]. The translocation causes formation of a hybrid *bcr-abl* gene and corresponding protein Bcr-Abl, which participates in the pathological process [3]. Our previous work showed the successful application of the oligonucleotide mod-Ph, whose nucleotide sequence is complementary to that region of the hybrid messenger RNA (mRNA), where exon 14 of the *bcr* gene and exon 2 of the *abl* gene are juxtaposed, for preparation of bioselective element of the biosensor based on surface plasmon resonance (SPR) spectrometer “Plasmon SPR-4m” [4]. Specific hybridization between immobilized mod-Ph and complementary oligonucleotide P1 was detected in label-free and real-time measurements, whereas no sensor response to non-complementary target was observed. The further investigations of selectivity of the developed biosensor have led to the necessity of the discrimination of completely complementary and partially complementary oligonucleotides corresponding to hybrid and normal (non-translocated) nucleotide sequences, respectively.

Discrimination of completely complementary and partially complementary DNA duplexes on the sensor surface can be achieved by employing various factors influencing hybridization efficiency, including target length and concentration, temperature, composition of hybridization buffer, posthybridization stringency wash, and a combination of the factors [5, 6]. The choice of stringency control strategies depends on various factors, including the sensing platform used. So, the application of hybridization suppressors like formamide with a high refractive index, which is out (or nearly out) of the detectable range of most SPR equipments, results in a technical challenge for SPR detection of DNA hybridization [5–9].

Unlike hybridization suppressors, stringency control based on the ionic strength of the hybridization buffer, which strongly influences hybridization of completely complementary and partially complementary DNA duplexes and does not lead to excessive strong refractive index change, appears quite attractive [10]. For SPR spectrometry-based DNA analysis, employment of high temperature is not generally recommended because the refractive index sensitivity of SPR is influenced by temperature and high temperature will generate considerable complications in SPR responses [7]. Moreover, not all SPR equipments have temperature control functions. However, we developed a SPR spectrometer measuring cell with built-in high-precision temperature control and regulation, able to heat the solution, which is pumped through the measuring cell, up to 65 °C and determine its value with an accuracy of 0.1 °C.

The aim of this research was to detect and discriminate the oligonucleotides related to the *bcr-abl* hybrid mRNA from those related to the normal *bcr* gene using two stringency control strategies based on the decrease in ionic strength of the hybridization buffer and the temperature elevation by SPR spectrometry biosensor.

## Methods

### Reagents

Urea, KH_2_PO_4_, 6-mercapto-1-hexanol was obtained from Fluka (Buchs, Switzerland); all other reagents were of analytical grade. All solutions were made with deionized MilliQ water.

Table 1 identifies the oligonucleotides used in these experiments. The HPLC-purified single-stranded oligonucleotide probe, functionalized at the 5′-end with a thiol group connected by a hexamethylene linker (HS-(CH2)6-ssDNA), and HPLC-purified oligonucleotide targets were obtained from Metabion International AG (Germany). The choice of the length and nucleotide sequence of the oligonucleotides used was described elsewhere [4].

**Table 1:** Oligonucleotide sequences and nomenclature

	Name	Sequence
Probe	Mod-Ph	HS- (CH_2_)_6_ -5′- GCT GAA GGG CTT TTG AAC TCT GCT-3′
Target	P1	5′- AGC AGA GTT CAA AAG СCC TTC AGC-3′
Target	Bcrex14	5′- CCA CTG GAT TTA AGC AGA GTT CAA-3′
Target	TC	5′- GCT ATC AGC CAC GAA CAC CCA-3′

### Immobilization of Thiol-Modified Oligonucleotides on the Sensor Surface and Their Hybridization with Target Oligonucleotides

To investigate the processes of oligonucleotide immobilization and hybridization, we used the two-channel SPR spectrometer “Plasmon SPR-6.” This computer-controlled optoelectronic device in the Kretschmann optical configuration was developed at the V.E. Lashkarev Institute of Semiconductor Physics of the National Academy of Sciences of Ukraine. A 45-nm-thick gold layer on a glass plate serves as a sensor surface. Specific recognition layer on the sensor surface (bioselective element) is prepared as follows. Prior to modification, the gold surface of the glass plate is cleaned with freshly prepared piranha solution (3:1 mixture of concentrated H_2_SO_4_ and 30 % H_2_O_2_; WARNING: *piranha solution reacts violently with organic compounds and must be handled with extreme care*) at room temperature for 2 min, then rinsed thoroughly with distilled and deionized water, and dried in the air. The cleaned plate is mounted on the spectrometer prism using immersion liquid. The flow rate (usually 40 μl min^−1^) is controlled by the peristaltic pump “Ismatec.” For immobilization of the thiolated probe, 120 μl 1 μM mod-Ph in 0.5 M KH_2_PO_4_ (pH 3.8) is injected into the measuring flow cell and exposed for 1 h. After that, the sites on the gold surface, free from immobilized mod-Ph, were blocked by 1 mM aqueous solution of 6-mercapto-1-hexanol [11].

At the beginning of the hybridization experiment, the measuring flow cell is thoroughly washed by the running buffer solution, for example, 2 × SSC (30 mM NaCitrate, 300 mM NaCl, pH 7) to obtain a stable sensor signal. At the next step, 120 μl target solution of various concentrations in the running buffer solution is injected into the measuring flow cell and exposed for 10 min. To distinguish an actual sensor response caused by interactions between the target and the immobilized probe from that caused by occasional fluctuations of medium refractive index, it is necessary to wash the flow cell before and after each sample by the same buffer solution and only then to determine a value of the SPR response. Regeneration of the bioselective element after hybridization experiments was achieved by denaturation of the surface duplexes by rinsing with 8 M urea.

## Results and Discussion

We have chosen a sequence consisting of 12 nucleotides of the *bcr* gene and 12 nucleotides of the *abl* gene of the site of their junction in the hybrid mRNA as a target oligonucleotide P1 for the sensor development. Therefore, completely complementary oligonucleotide mod-Ph is used as a probe for immobilization on the sensor surface.

The chosen conditions (1 μM mod-Ph in 0.5 M KH_2_PO_4_, pH 3.8) have led to an efficient immobilization of the probe on the gold surface of the SPR biosensor. The changes in the resonance angle at the mod-Ph immobilization were converted into surface density using a factor of 120 mdeg per 100 ng cm^−2^ [12, 13], and it was equal to about 92 ng cm^−2^ (7.6 × 10^12^ molecules cm^−2^). The theoretical coverage of a two-dimensional close-packed full monolayer of single-stranded DNA (ssDNA) is 1 × 10^14^ molecules cm^−2^, assuming that the molecules are perfect cylinders 1.1 nm in diameter and are oriented perpendicularly to the plane of the surface [14]. Using this calculation in our case, the coverage represents about 8 % of a saturated monolayer for thiolated ssDNA. Herne and Tarlov [11] assumed that very high surface coverage of ssDNA does not allow the optimal hybridization efficiency as the complementary sequence can be sterically prevented from hybridizing at high surface DNA density. In this work, we obtained an efficient but far from saturated immobilization of mod-Ph on the gold surface of the SPR biosensor.

**Fig. 1:**
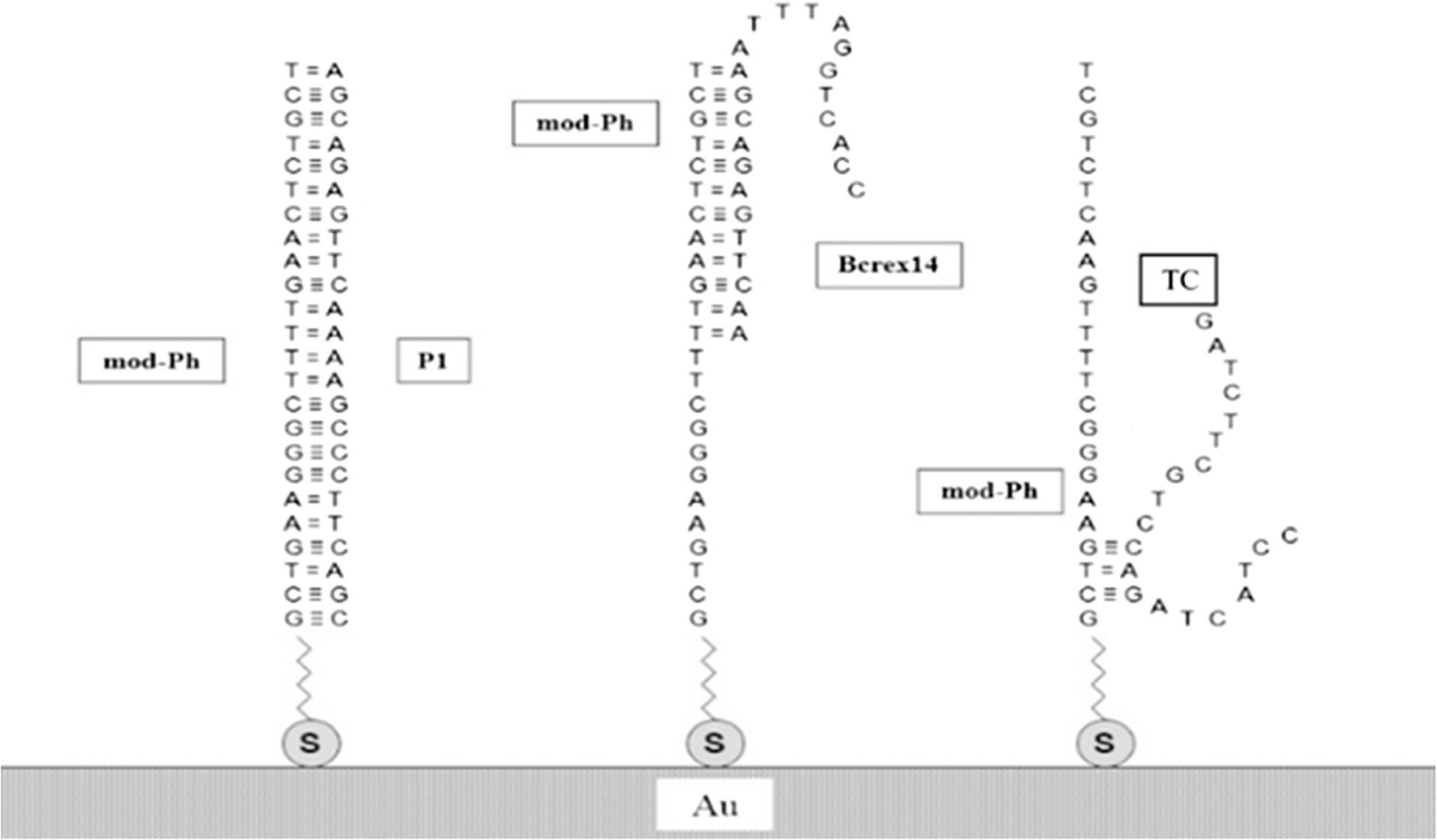
The most likely intermolecular duplexes forming at hybridization of the targets P1, Bcrex14, and TC with the immobilized probe mod-Ph

To study selectivity of the developed biosensor, along with completely complementary to the probe mod-Ph target oligonucleotide P1, two other oligonucleotides were used: practically non-complementary TC and partially complementary Bcrex14, which was derived from the normal gene *bcr*. Using the web server DINAMelt [15, 16], we obtained the Gibbs energy values of the solution-phase hybridization between the probe mod-Ph and the targets P1, Bcrex14, and TC (Table 2) and the most likely intermolecular duplex structures forming at hybridization of the above oligonucleotides Fig. 1.

**Table 2:** Gibbs energy of the solution-phase hybridization between the probe mod-Ph and the targets P1, Bcrex14, and TC depending on the buffer stringency

Probe and target	ΔG (kcal/mol)		
	0.5 × SSC	1 × SSC	2 × SSC
Mod-Ph and P1	−32.2	−33.9	−35.6
Mod-Ph and Bcrex14	−13.9	−14.7	−15.6
Mod-Ph and TC	−4.0	−4.3	−4.6

These values show that an increase in the ionic strength leads to gradual strengthening of intermolecular interactions that can be explained by shielding of negatively charged phosphate residues of oligonucleotides by counterions of the buffer solution and, consequently, creating more favorable conditions for hydrogen bonding between complementary nucleic bases.

The obtained values for the case of the 2 × SSC buffer solution allow supposing that the probe mod-Ph strongly interacts with the completely complementary P1, can interact with partially complementary Bcrex14, and does not interact with the non-complementary TC.

**Fig. 2:**
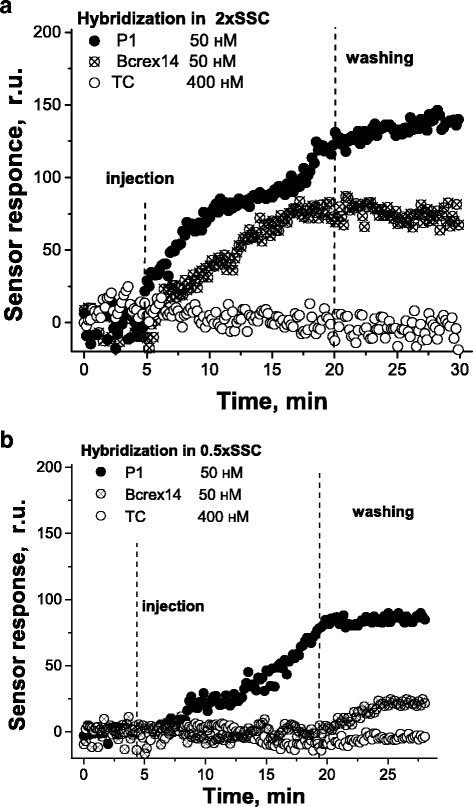
SPR sensograms for hybridization between the immobilized probe mod-Ph and the targets P1, Bcrex14, and TC in 2 × SSC (**a**) and in 0.5 × SSC (**b**)

The experimental SPR responses shown in Fig. 2a and obtained in the 2 × SSC buffer solution are in good agreement with the theoretical predictions mentioned above. Indeed, P1 shows the highest sensor response, Bcrex14 produces a smaller response, and no response was observed at injection of even much higher concentration of the non-complementary oligonucleotide TC. It should be noted that the sensor signal reached after injection and incubation of P1 or Bcrex14 did practically not decrease during the washing by the running buffer solution. It confirms that stable duplexes are formed on the sensor surface.

Under higher stringency conditions (a lower buffer concentration, in this case, 0.5 × SSC), the reduced level of hybridization can be observed for both targets, but for the less complementary oligonucleotides, it was somewhat much stronger (Fig. 2b). The ratio of the sensor response at the P1/mod-Ph hybridization to the sensor response at the Bcrex14/mod-Ph hybridization can serve as an indicator of the level of discrimination or discrimination index of completely complementary and partially complementary targets.

**Fig. 3:**
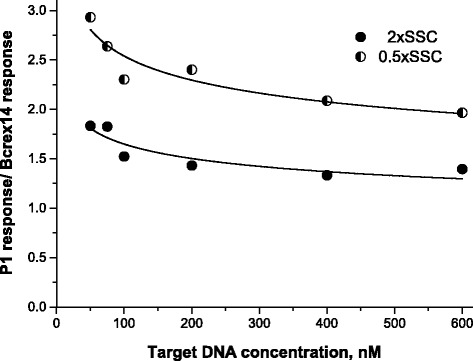
Dependence of the ratio of the sensor response at the P1/mod-Ph hybridization to the sensor response at the Bcrex14/mod-Ph hybridization on the concentration of the targets in two buffer solutions used

Figure 3 demonstrates obvious advantages of the use of a higher stringency condition (0.5 × SSC): depending on the target concentration, the values of the ratio of the sensor response at the P1/mod-Ph hybridization to the sensor response at the Bcrex14/mod-Ph hybridization varied from 1.3 to 1.8 in 2 × SSC and from 2.0 to 2.9 in 0.5 × SSC. For comparison, Lao and coworkers [9] reported that under their optimal conditions (using formamide), a mismatched 22-mer was detected with 1.7 and 2.8 times less hybridization signals compared with the perfectly matched oligonucleotide, for DNA probe and peptide nucleic acid probe, respectively. Thus, the use of 0.5 × SSC allowed comparably pronounced discrimination of the completely complementary and partially complementary oligonucleotides related to the hybrid *bcr-abl* and to the normal *bcr* genes.

Using the web server DINAMelt [15, 16], we obtained the melting temperature (*T*_m_) values (the temperature, which corresponds to state when half of DNA duplexes are melted) of the solution-phase hybridized duplexes of the probe mod-Ph and the targets P1 and Bcrex14 (Table 3). A considerable difference of these values allows to suppose that controlling temperature in the measuring flow cell one can discriminate these completely and partially complementary oligonucleotides.

**Table 3:** The *T*_m_ values of the solution-phase hybridized duplexes of the probe mod-Ph and the targets P1 and Bcrex14 of the solution-phase determined using the web server DINAMelt and the experimental *T*_m_ values of the same duplexes formed on the sensor surface

Probe and target	*T*_m_ calculated (°C)	*T*_m_ experimental (°C)
Mod-Ph and P1	64.0	64.0
Mod-Ph and Bcrex14	39.2	39.2

These data indicate that in solution at room temperature, both pairs of the studied oligonucleotides form stable duplexes, and when the temperature rises to about 40 °C, majority of the duplexes mod-Ph/Bcrex14 melts, while duplexes mod-Ph/P1 remain stable. The heterogeneous system (with a solid sensor surface, on which oligonucleotide probes are immobilized) creates less favorable conditions for hybridization, and the corresponding values of *T*_m_ should be somewhat smaller. Nevertheless, one can assume with a high probability that with a gradual temperature elevation among these two pairs of oligonucleotides, mod-Ph/Bcrex14 duplexes will melt first.

This approach using SPR spectrometer can be realized as follows: (1) a level of sensor response corresponding to hybridization of the immobilized oligonucleotide probes with target oligonucleotides in the linear range SPR biosensor at the initial (room) temperature should be determined and (2) SPR biosensor measuring cell should be washed by the buffer solution at a specified elevated temperature for a certain period of time (in this work—5 min). As a result of this procedure, certain duplexes will be melted and the buffer solution flowing through the measuring cell will wash out the separated target oligonucleotides. After this, washout and subsequent cooling of the measuring cell to the initial temperature, sensor response, which will reflect the amount of the remaining duplexes should be determined.

Successful application of the thermodiscrimination is not possible without the immobilization stability of the surface-bound thiolated probes at the elevated temperature. However, the wide application of the regeneration of the single-stranded thiolated probe layer after hybridization experiments by rinsing with hot water (> 60 °C) proves the stability of the probe layer [11, 17, 18]. Our highly reproducible hybridization data confirm also the sufficient thermostability of the immobilized mod-Ph.

**Fig. 4:**
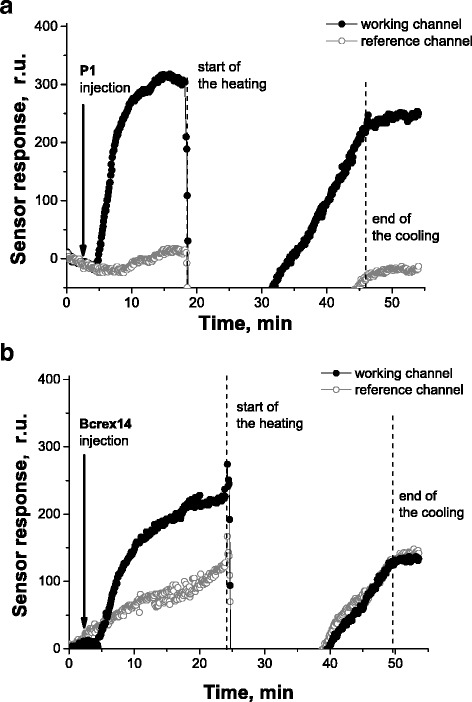
Discrimination of P1 (**a**) and Bcrex14 (**b**) using controlled temperature change in the measuring cell of the SPR spectrometer “Plasmon 6”

For implementation of the thermodiscrimination, thermoregulated measuring flow cell for the SPR spectrometer “Plasmon 6” and corresponding software were created. It is able to ensure rapid heating of the buffer solution that is pumping through the measuring cell to the desired temperature and control it to within 0.1 °C. In addition, it can clearly trace cooling of the buffer solution to determine the return to the initial temperature. This device allowed to carry out thermodiscrimination of completely complementary oligonucleotide P1 and partially complementary oligonucleotide Bcrex14, an example of which is shown in Fig. 4.

In this example, the difference between working and reference channels during oligonucleotide hybridization before heating, during heating, and after cooling to the original level of temperature was monitored. The success of the discrimination was provided by significant difference between sensor responses, caused by processes of hybridization/dehybridization of these two pairs of oligonucleotides. After heating to 40 °C, the duplexes formed by partially complementary oligonucleotide Bcrex14 were melted and almost completely washed out from the measuring cell (SPR signal returned to the level of the signal observed before Bcrex14 injection into the measuring cell). In the case of complementary oligonucleotide P1, the opposite situation was observed: at the same procedure of hybridization, heating temperature and cooling the difference of sensor responses of working and reference channels was near the same as that, which was observed at the initial hybridization.

The ratio of these sensor responses is the analytical indicator that shows the remaining portion of the duplexes, formed by various oligonucleotides. And, its dependence on temperature for different pairs of oligonucleotides shows that temperature range, in which thermodiscrimination of completely and partially complementary oligonucleotides could be carried out.

**Fig. 5:**
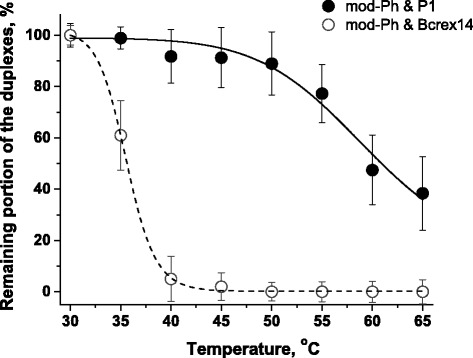
The dependence of the ratio of sensor response after hybridization of target oligonucleotide P1 or Bcrex14 with immobilized mod-Ph, heating and cooling to the initial sensor response on the heating temperature

As shown in Fig. 5, the duplexes formed by completely complementary oligonucleotide P1 (and mod-Ph) are remained without significant changes until ~50 °C, while the duplexes formed by partially complementary oligonucleotide Bcrex14 (and mod-Ph) since 40 °C almost entirely disrupted. Thus, one could say that the thermodiscrimination of this pair of completely and partially complementary oligonucleotides is absolutely effective at the temperature from ~40 to ~50 °C.

Besides that, these experimental data allow to calculate the experimental values of *T*_m_. To obtain these values, a sigmoid fitting function was used and the corresponding values have been taken from the curves from Fig. 5. As it was expected, the experimental values of *T*_m_ determined for heterogeneous system (hybridization on the sensor surface) are a few degrees lower than the values obtained using the web server DINAMelt for the solution-phase hybridization (Table 3). Melting of a nucleic acid duplex in solution means total unzipping of the duplex, where the individual strands can freely move away from each other. The same pathway is not expected to be followed within a film at a solid–liquid interface since each duplex is closely surrounded by a number of other duplexes within a film structure. The local concentration of the negatively charged oligonucleotides and therefore the probe density could be considerably higher on the surface compared to that in bulk solution since the probes can be chemically anchored on the surface. This allows both the steric effects and the electrostatic effects to play an important role in solid-phase hybridization. Moreover, as the melting reaction proceeds to achieve the equilibrium between the solution and the probe concentrations on the gold surface, the removal of the solution after each heating step could cause denaturation of some more duplexes even if at the same temperature. So, the accurate *T*_m_ values of the surface-confined duplexes would be somewhat higher than the *T*_m_ values measured herein [18].

## Conclusion

We report the results of the application of two stringency control strategies for SPR detection of DNA hybridization and discrimination of completely and partially complementary oligonucleotides related to the human normal *bcr* and hybrid *bcr-abl* genes.

The development of temperature-independent stringency control for SPR DNA detection and discrimination (for example, based on decreasing the ionic strength of hybridization buffer solution) is of importance because of technical simplicity of its realization, but it produced a relatively moderate level of discrimination and therefore requires a careful accounting of the impact of possible high concentrations of partially complementary DNA.

For implementation of the thermodiscrimination, thermoregulated measuring flow cell for the SPR spectrometer Plasmon 6 and corresponding software were created. Using this thermoregulated measuring flow cell allowed the absolutely effective thermodiscrimination of the completely complementary oligonucleotide P1 and the partially complementary oligonucleotide Bcrex14.

Besides that, this approach gives the opportunity to calculate the experimental values of *T*_m_ of the duplexes formed by the studied oligonucleotides. As it was expected, the experimental values of *T*_m_ determined for the heterogeneous system (hybridization on the sensor surface) are a few degrees lower than the values obtained using the web server DINAMelt for the solution-phase hybridization.

It is necessary to note that this method of thermodiscrimination is very flexible because the developed device can control the temperature to within 0.1 °C and a time of hot wash can be selected almost without restriction. In general, we believe the obtained results (especially those obtained due to the stringency control based on the temperature elevation) are very important for the future development of the SPR biosensor for clinical detection of the *bcr-abl* gene related to some types of leukemia.
